# Myopia Control in Caucasian Children with 0.01% Atropine Eye Drops: 1-Year Follow-Up Study

**DOI:** 10.3390/medicina60071022

**Published:** 2024-06-21

**Authors:** Dovile Simonaviciute, Arvydas Gelzinis, Laura Kapitanovaite, Andrzej Grzybowski, Reda Zemaitiene

**Affiliations:** 1 Department of Ophthalmology, Medical Academy, Lithuanian University of Health Sciences, 44037 Kaunas, Lithuania; 2 Department of Ophthalmology, Hospital of Lithuanian University of Health Sciences, Kaunas Clinics, 50161 Kaunas, Lithuania; 3 Department of Ophthalmology, University of Warmia and Mazury, 10-724 Olsztyn, Poland; 4 Institute for Research in Ophthalmology, 60-554 Poznan, Poland

**Keywords:** myopia control, myopia progression, low-dose atropine eye drops

## Abstract

*Background and Objectives*: Myopia is the most widespread ocular disorder globally and its prevalence has been increasing over the past decades. Atropine eye drops stand out as the only pharmacological intervention used in clinical practice to control myopia progression. The aim of this study was to explore the effect of 0.01% atropine eye drops on myopia progression. *Patients and Methods*: Healthy children aged 6–12 years with cycloplegic spherical equivalent (SE) from −0.5 D to −5.0 D and astigmatism ≤1.5 D were included. Myopia progression was assessed by changes in SE and axial length (AL) over 1 year and SE changes 1 year before the study enrollment and during the 1-year follow-up. Adverse events were evaluated based on complaints reported by either parents or the children themselves during follow-up visits. *Results*: The analysis involved 55 patients in the 0.01% atropine eye drops group and 66 in the control group. After the 1-year follow-up, the change in SE was −0.50 (−2.25–0.50) D in the control group compared to −0.50 (−1.50–0.50) D in the 0.01% atropine group (*p* = 0.935); AL change was 0.31 (0.18) mm in the control group and 0.29 (0.18) mm in the 0.01% atropine group (*p* = 0.480). The change in SE was −0.68 (−2.0–−0.25) D/year before the study and remained similar −0.50 (−2.25–0.25) D over the 1-year follow-up in the control group (*p* = 0.111); SE change was reduced from −1.01 (−2.0–−0.25) D/year before the study to −0.50 (−1.5–0.5) D over the 1-year follow-up in the 0.01% atropine group (*p* < 0.001). In the 0.01% atropine group, ten (16.4%) children experienced mild adverse events, including blurred near vision, ocular discomfort, photophobia, dry eyes, and anisocoria. *Conclusions*: Compared to the control group, the administration of 0.01% atropine eye drops demonstrated no significant effect on changes in SE and AL over a 1-year follow-up. However, children in the 0.01% atropine group initially experienced higher myopia progression, which decreased with treatment over the course of 1 year. Future studies should explore the long-term effects, rebound effects, potential genetic associations, and efficacy of higher doses of atropine in managing myopia progression.

## 1. Introduction

Myopia is the most prevalent ocular disorder globally [1]. It is predicted that approximately half of the world’s population will be myopic by 2050, of which 10% will have a high degree of myopia [1]. High myopia is a risk factor for sight-threatening complications [2]. Therefore, myopia progression control has become one of the most popular topics in ophthalmic research.

Among the measures employed to slow myopia progression, atropine eye drops have emerged as the only pharmacological intervention implemented in clinical practice [3,4]. Atropine has been reported to have a dose-dependent effect on myopia progression at the cost of increasing the risk of adverse events and rebound effects at higher concentrations [5,6,7]. Studies confirmed the efficacy of higher concentrations of atropine eye drops (0.5–1.0%) for myopia control, but side effects and rebound after drop cessation frequently occurred [5,8]. In more recent research, the efficacy of low-concentration atropine eye drops (0.01–0.05%) has been confirmed, with fewer side effects and better compliance [6,9,10,11]. Furthermore, 0.01% atropine is commonly regarded as the preferred initial concentration. However, its effect on myopia progression and the optimal treatment strategy remains uncertain [7,10,11,12,13,14,15,16]. In this study, we explore the effect of 0.01% atropine eye drops on myopia progression in a homogenous population of Caucasian children over the period of 1 year.

## 2. Patients and Methods

### 2.1. Study Design

A prospective study on 0.01% atropine efficacy was conducted at the Department of Ophthalmology of the Hospital of Lithuanian University of Health Sciences, Kaunas clinics from March 2021 to July 2023. Informed consent from at least one parent or legal guardian and verbal assent from the child were obtained. The procedures adhered to the Declaration of Helsinki, and approval from the Lithuanian Bioethics Committee was obtained (No. BE-2-18).

### 2.2. Study Population

Caucasian healthy children aged 6 to 12 years with cycloplegic spherical equivalent (SE) (sphere plus half of the cylinder power) refraction from −0.5 D to −5.0 D and astigmatism of 1.5 D or less were included. All myopic children enrolled in the study were advised to undergo treatment with 0.01% atropine eye drops. For the control group, we included patients who consented to participate in the study but refused to be treated with 0.01% atropine eye drops. The atropine group consisted of children who agreed to use 0.01% atropine eye drops once nightly in both eyes for one year. Patients were required to purchase the atropine eye drops at their own expense from a pharmacy that provides drug compounding services. Key exclusion criteria included previous use of atropine eye drops or other methods for myopia progression slowdown, congenital or chronic ocular disease, anisometropia greater than 1.5 D, history of ocular surgeries, systemic diseases, or chromosomal abnormalities.

### 2.3. Procedures

A comprehensive ocular examination of myopic children was conducted. All measurements were taken at the baseline and after 12 months, focusing on the right eye for analysis. Cycloplegic refraction was measured using the autorefractor TonoRef III (Nidek Co., Ltd., Gamagori, Japan) after administering 1% cyclopentolate twice, with a 5 min interval. Refraction was evaluated 20 min after the second drop. Ocular axial length (AL) was measured using the partial coherence interferometer IOLMaster 700 (Carl Zeiss Meditec AG, Jena, Germany). For those patients who had previously visited our clinic for an ophthalmological examination, myopia progression before study enrollment was assessed. Single-vision spectacle lenses were prescribed to all the patients.

### 2.4. Outcomes

The primary outcomes of the study were the change in mean SE under cycloplegia and AL from baseline to the 1-year mark. Initially, data were compared between the control group and 0.01% atropine eye drops group. Secondary outcomes included the change in SE 1 year before enrollment to the study and over the 1-year follow-up. Tolerability and adverse events were evaluated through complaints reported by the parents or the children themselves during follow-up visits.

### 2.5. Statistical Analysis

Statistical analyses were conducted using SPSS version 29. The distribution of the data was assessed using Kolmogorov–Smirnov or Shapiro–Wilk tests. The comparison between the groups was conducted using an independent-sample *t*-test or the Mann–Whitney U test. The Wilcoxon rank sum test was used to compare myopia progression before study enrollment and over 1 year. Categorical data were analyzed using the Chi-square test. The average time in hours of outdoor activities (during spring/summer and autumn/winter seasons) and near-work time (homework and reading), screen time (mobile phone, computer, and TV), and sleeping were collected, as well as the self-reported age at myopia onset, weight and height, and number of parents with myopia. To calculate the average time spent on outdoor activities, near-work, and screen time, we employed the following formula: (hours spent on weekday × 5/7) + (hours spent on weekend day × 2/7). The questionnaire was completed by the parents. The results are presented as counts (frequencies), mean (standard deviation), or median (minimal–maximal) values. Statistical significance was set at *p* < 0.05.

## 3. Results

There were 75 patients in the 0.01% atropine group and 84 patients in the control group. Over the course of 1 year, 20 patients from the 0.01% atropine group and 18 patients from the control group withdrew from the study. In the 0.01% atropine group, seven participants were excluded due to difficulties adhering to eye drops regime, six participants discontinued the eye drops because of adverse events (ocular discomfort (two patients), blurred near vision (three patients), and photophobia (one patient)), five were lost to follow-up, one received additional myopia treatment, and one was diagnosed with systemic lupus erythematosus. In the control group, 17 patients were lost to follow-up and 1 received alternative myopia treatment. The final analysis included 55 patients in the 0.01% atropine eye drops group and 66 patients in the control group.

No significant differences were observed in age, gender, baseline AL, baseline SE, self-reported age at diagnosis of myopia, body mass index (BMI), parental myopia, and lifestyle features between the 0.01% atropine group and the control group (Table 1).

### 3.1. Efficacy Outcomes

The median change in cycloplegic SE from baseline to 1 year was −0.50 (−2.25–0.50) D in the control group and −0.50 (−1.50–0.50) D in the 0.01% atropine eye drops group (*p* = 0.935). The change in mean AL from baseline to 1 year was 0.31 (0.18) mm in the control group and 0.29 (0.18) mm in the 0.01% atropine group (*p* = 0.480).

Thirty-four patients in the control group and 45 in the 0.01% atropine eye drops group visited our clinic for an ophthalmological examination before study initiation. The median age was 10 (7–12) years in the control group and 10 (6–12) years in the 0.01% atropine group (*p* = 0.831). Among these patients, the baseline SE was −2.29 (0.85) D in the control group and −2.66 (1.20) D in the 0.01% atropine eye drops group (*p* = 0.115) and the baseline AL was 24.18 (1.0) mm and 24.53 (0.67) mm (*p* = 0.085), respectively. Analyzing the changes in SE during the 1-year period preceding study enrollment, the median change in SE was −0.68 (−2.0–−0.25) D/year in the control group, compared to −1.01 (−2.0–−0.25) D/year in the atropine group (*p* = 0.018) (Table 2). SE changes of −0.5 D or less were observed in 8 (17.8%) cases in the 0.01% atropine eye drops group compared to 14 (41.2%) cases in the control group (*p* = 0.022). Notably, children in the 0.01% atropine eye drops group experienced significantly greater myopia progression before study enrollment compared to the control group. In the control group, myopia progression remained similar over the 1-year follow-up (*p* = 0.111), while it decreased significantly from −1.01 (−2.0–−0.25) D to −0.50 (−1.5–0.5) D in the 0.01% atropine group (*p* < 0.001) compared to the progression observed before study enrollment.

### 3.2. Safety Outcomes

Ten (16.4%) children experienced mild adverse events in the 0.01% atropine group, with six (9.8%) of them discontinuing the use of 0.01% atropine eye drops. Blurred near vision (four patients, 6.6%), ocular discomfort (three patients, 4.9%), photophobia (one patient, 1.6%), dry eyes (one patient, 1,6%), and anisocoria (one patient, 1.6%) were the chief complaints. None of the adverse events were considered severe.

## 4. Discussion

In the present study, the effect of 0.01% atropine eye drops on myopia progression in a real-life clinical setting among children aged 6–12 years, with SE ranging from −0.5 to −5.0 D, was investigated. The comparison of changes in SE and AL between the control group and 0.01% atropine eye drops group revealed that the use of 0.01% atropine eye drops did not significantly affect myopia progression over the 1-year follow-up period. However, when analyzing changes in SE 1 year before study enrollment and during the 1-year follow-up, we observed a decrease in myopia progression from −1.01 (−2.0–−0.25) D/year to −0.50 (−1.5–0.5) D/year in the 0.01% atropine group (*p* < 0.001). Although no significant difference was found between the 0.01% atropine eye drops group and the control group regarding changes in SE and AL over the 1-year follow-up, it is notable that the atropine group experienced greater myopia progression prior to enrollment, which decreased with treatment over the course of 1 year. This suggests the potential role of 0.01% atropine eye drops in slowing down myopia progression, especially considering that SE changes remained similar in the control group.

Numerous studies have confirmed the efficacy of 0.01% atropine eye drops in slowing myopia progression. For instance, a 5-year Spanish study involving children aged 9–12 with myopia ranging from −0.5 to −2.0 D reported a mean annual myopia progression of −0.14 (0.35) D in the 0.01% atropine group compared to −0.65 (0.54) D in the control group (*p* < 0.01) [17]. Notably, it exclusively included children aged 9–12 with myopia ranging from −0.5 to −2.0 D showing better efficacy of 0.01% atropine compared to this study’s findings. Another Spanish study by M. Moriche-Carretero et al. [18] demonstrated reduced myopia progression of −0.51 (0.39) D in the 0.01% atropine group compared to −0.76 (0.37) D in the control group over a 2-year follow-up, with AL increasing by 0.20 (0.20) mm and 0.37 (0.27) mm, respectively (*p* < 0.001).

In contrast, a study conducted in Ireland by Loughman et al. [19] enrolled children aged 6 to 16 years with SE ≤ −0.50 D and either 0.01% atropine or placebo eye drops were prescribed for 2 years. The majority of participants were white (80.8% in the 0.01% atropine group and 86.7% in the placebo group). The difference in changes in SE between the 0.01% and placebo groups was not significantly different at the 2-year visit (*p* = 0.07) but it was notably lower in the atropine group at the 18-month visit (*p* = 0.049). Additionally, AL elongation was reduced in the atropine group at both the 18-month (*p* = 0.04) and 24-month visits (*p* = 0.009) compared to the placebo group. At the 1-year visit, there were no statistically significant differences observed between the groups in terms of changes in SE and AL. An analysis based on ethnicity revealed that 0.01% atropine eye drops were more effective at reducing SE and AL changes in white participants compared to non-white participants: statistically significant differences in SE and AL changes were found at the 18-month and 2-year visits in white participants, whereas no statistically significant difference was found in non-white participants at any study point. A randomized clinical trial conducted in the USA involving children of mixed ethnicities revealed that in comparison to the placebo group, the use of 0.01% atropine eye drops did not effectively attenuate myopia progression based on changes in SE and AL after two years of treatment, and the authors do not support the use of 0.01% atropine eye drops to slow myopia progression [13]. Another study involving a multi-racial cohort of Australian children aged 6 to 16 years with SE of −1.50 D or less found a statistically significant difference between the 0.01% atropine and placebo group at 1 year but not at the 2-year follow-up: the mean SE and AL change from baseline to 1 year were −0.31 D (95%CI = −0.39 to −0.22) and 0.16 mm (95%CI = 0.13–0.20) in the 0.01% atropine group and −0.53 D (95%CI= −0.66 to −0.40) and 0.25 mm (95%CI = 0.20–0.30) in the placebo group (group difference *p* ≤ 0.01); at 2 years, SE and AL were −0.64 D (95%CI = −0.73 to −0.56) and 0.34 mm (95%CI = 0.30–0.37) in the 0.01% atropine group and −0.78 D (95%CI = −0.91 to −0.65) and 0.38 mm (95%CI = 0.33–0.43) in the placebo group, respectively (group difference *p* = 0.10) [20]. The authors proposed that the increased number of participants lost to follow-up in the placebo group, particularly those with more rapid myopia progression, could have influenced the inability to detect a treatment effect at 2 years.

Different clinical and demographic factors, such as baseline rates of progression, degree of myopia, age at presentation of myopia and ethnicity, may be associated with myopia progression [20]. While the results of this study revealed the effectiveness of 0.01% atropine eye drops when analyzing SE progression 1 year before the beginning of the study and over the 1-year follow-up in 0.01% atropine group, no significant differences in AL and SE changes were observed between the 0.01% atropine eye drops group and the control group over the study period. This lack of distinction in our study may be attributed to the possibility that a significant proportion of participants in the atropine group experienced greater myopia progression prior to the initiation of the study. Additionally, since parents had the option to choose whether to begin treatment with 0.01% atropine eye drops or not, the parents of children with more progressive myopia were more likely to start the treatment. Furthermore, a duration of one year may not be sufficiently long to detect differences, as some studies suggest that the greater efficacy of 0.01% atropine eye drops may manifest in the second year [19,21,22]. However, neither the study conducted in the USA [13] nor the study performed in Australia demonstrated the efficacy of 0.01% atropine in controlling myopia progression after 2 years of use [20].

Regarding adverse reactions, blurred near vision was the primary complaint in the 0.01% atropine eye drops group, affecting four patients. In total, ten patients (16.4%) reported complaints related to the atropine eye drops. The frequency of adverse events in this study is similar to that in other studies conducted in non-Asian regions using 0.01% atropine eye drops [11,16,23]. Joachimsen et al. analyzed the differences in side effects between 0.01% and 0.05% atropine eye drops in myopic German schoolchildren [24]. They found that children treated with 0.05% atropine eye drops exhibited significantly higher anisocoria and a greater loss of accommodation amplitude compared to children treated with 0.01%. Side effects were more pronounced in Caucasians treated with 0.05% atropine eye drops compared to reported side effects in Asian children. In this study, the choice of the lowest concentration of 0.01% was made to minimize the risk of adverse events and to achieve the best patient compliance.

### Strengths and Limitations

The notable strength of this study lies in its significant representation of Caucasian children, comprising 100% of the study population. This stands out as the majority of research on low-concentration atropine has predominantly focused on Asian children.

However, the current research does have several important limitations. Firstly, we did not have access to data regarding the extent of myopia progression for all the patients prior to their enrollment in the study, nor did we possess information on AL measurements before study initiation. Secondly, because parents were given the choice to decide whether to use atropine eye drops, those with children exhibiting more progressive myopia were more likely to begin the treatment. Thirdly, we were not able to assess patient compliance with the treatment regimen and patients had to purchase the drops at their own expense at local pharmacies. Moreover, this study focused exclusively on a 0.01% atropine concentration. The focus of our upcoming studies includes exploring higher concentrations of atropine eye drops and assessing the significance of genetic factors in controlling the progression of myopia.

## 5. Conclusions

In conclusion, the data from the present study show that the effect of 0.01% atropine eye drops on myopia progression still remains controversial. Over the course of a 1-year follow-up, the administration of 0.01% atropine eye drops demonstrated no significant effect on changes in SE and AL compared to the control group. However, it was observed that the participants in the 0.01% atropine eye drops group exhibited greater myopia progression prior to study enrollment, which appeared to be attenuated over the 1-year period with treatment. Future studies should explore long-term effects, rebound effects, potential genetic associations, and the impact of higher atropine doses in managing myopia progression.

## Figures and Tables

**Table 1:** Baseline data distribution of the study population.

Variables	Control Group (*n* = 66)	0.01% Atropine Group (*n* = 55)	*p*-Value
Gender: Male/female	23/43	19/36	0.972
Baseline age, years	10 (6–12)	10 (6–12)	0.673
Baseline SE, D	−2.06 (−4.63–−0.88)	−2.38 (−5.0–−0.5)	0.071
Baseline AL, mm	24.16 (1.03)	24.46 (0.65)	0.053
Both parents are myopic, cases	18	10	0.193
Self-reported age at diagnosis of myopia, year	9 (5–12)	8 (5–12)	0.278
BMI, kg/m^2^	18.11 (3.82)	17.27 (2.32)	0.440
Sleeping time, hours	9 (7.5–11)	9 (7–11)	0.882
Near-work time, hours	2.29 (0.96)	2.42 (0.85)	0.465
Screen time, hours	4.14 (1.0–12)	3.86 (1.0–13.0)	0.771
Time spent outdoor in spring-summer, hours	3.79 (1.0–8.43)	3.50 (1.36–8.0)	0.909
Time spent outdoor in autumn-winter, hours	1.5 (0.5–5.93)	1.5 (0.5–4.0)	0.905

Abbreviations: SE—spherical equivalent, D—diopter, AL—axial length, SD—standard deviation, BMI—body mass index.

**Table 2:** Changes in spherical equivalent in the control group and 0.01% atropine group 1 year before the study enrollment and over the 1-year follow-up.

Variables	Control Group (*n* = 34)	0.01% Atropine Eye Drops Group (*n* = 45)	*p*-Value
Change of SE 1-year before the study, D/year	−0.68 (−2.0–−0.25)	−1.01 (−2.0–−0.25)	0.018
Change of SE over 1-year follow-up, D/year	−0.50 (−2.25–0.25)	−0.50 (−1.5–0.5)	0.721
	*p* = 0.111 ^1^	*p* < 0.001 ^2^	

Abbreviations: SE—spherical equivalent, D—diopter; Note: ^1^ Comparison of SE progression 1 year before the study and during 1-year follow-up in the control group. ^2^ Comparison of SE progression 1 year before the study and during 1-year follow-up in the 0.01% atropine eye drops group.

## Data Availability

The data that support the findings of this study are available upon request from the corresponding author, D.S.

## References

[1] Holden B.A., Fricke T.R., Wilson D.A., Jong M., Naidoo K.S., Sankaridurg P., Wong T.Y., Naduvilath T., Resnikoff S. (2016). Global Prevalence of Myopia and High Myopia and Temporal Trends from 2000 through 2050. Ophthalmology.

[2] Ikuno Y. (2017). Overview of the complications of high myopia. Retina.

[3] Huang J., Wen D., Wang Q., McAlinden C., Flitcroft I., Chen H., Bao F., Zhao Y., Hu L., Li X. (2016). Efficacy comparison of 16 interventions for myopia control in children: A network meta-analysis. Ophthalmology.

[4] Chierigo A., Desideri L.F., Traverso C.E., Vagge A. (2022). The Role of Atropine in Preventing Myopia Progression: An Update. Pharmaceutics.

[5] Chia A., Chua W.H., Cheung Y.B., Wong W.L., Lingham A., Fong A., Tan D. (2012). Atropine for the treatment of childhood Myopia: Safety and efficacy of 0.5%, 0.1%, and 0.01% doses (Atropine for the Treatment of Myopia 2). Ophthalmology.

[6] Yam J.C., Zhang X.J., Zhang Y., Wang Y.M., Tang S.M., Li F.F., Kam K.W., Ko S.T., Yip B.H., Young A.L. (2022). Three-Year Clinical Trial of Low-Concentration Atropine for Myopia Progression (LAMP) Study: Continued Versus Washout: Phase 3 Report. Ophthalmology.

[7] Ha A., Kim S.J., Shim S.R., Kim Y.K., Jung J.H. (2022). Efficacy and Safety of 8 Atropine Concentrations for Myopia Control in Children: A Network Meta-Analysis. Ophthalmology.

[8] Chua W.H., Balakrishnan V., Chan Y.H., Tong L., Ling Y., Quah B.L., Tan D. (2006). Atropine for the Treatment of Childhood Myopia. Ophthalmology.

[9] Yam J.C., Jiang Y., Tang S.M., Law A.K.P., Chan J.J., Wong E., Ko S.T., Young A.L., Tham C.C., Chen L.J. (2019). Low-Concentration Atropine for Myopia Progression (LAMP) Study: A Randomized, Double-Blinded, Placebo-Controlled Trial of 0.05%, 0.025%, and 0.01% Atropine Eye Drops in Myopia Control. Ophthalmology.

[10] Tsai H.R., Chen T.L., Wang J.H., Huang H.K., Chiu C.J. (2021). Is 0.01% atropine an effective and safe treatment for myopic children? a systemic review and meta-analysis. J. Clin. Med..

[11] Pérez-Flores I., Macías-Murelaga B., Barrio-Barrio J. (2021). Multicenter Group of Atropine Treatment for Myopia Control (GTAM). A multicenter Spanish study of atropine 0.01% in childhood myopia progression. Sci. Rep..

[12] Chia A., Lu Q.S., Tan D. (2016). Five-Year Clinical Trial on Atropine for the Treatment of Myopia 2 Myopia Control with Atropine 0.01% Eyedrops. Ophthalmology.

[13] Repka M.X., Weise K.K., Chandler D.L., Wu R., Melia B.M., Manny R.E., Kehler L.A.F., Jordan C.O., Raghuram A., Summers A.I. (2023). Low-Dose 0.01% Atropine Eye Drops vs Placebo for Myopia Control: A Randomized Clinical Trial. JAMA Ophthalmol..

[14] Lanca C., Pang C.P., Grzybowski A. (2023). Effectiveness of myopia control interventions: A systematic review of 12 randomized control trials published between 2019 and 2021. Front Public Heal..

[15] Li Y., Yip M., Ning Y., Chung J., Toh A., Leow C., Liu N., Ting D., Schmetterer L., Saw S.-M. (2024). Topical Atropine for Childhood Myopia Control: The Atropine Treatment Long-Term Assessment Study. JAMA Ophthalmol..

[16] Sacchi M., Serafino M., Villani E., Tagliabue E., Luccarelli S., Bonsignore F., Nucci P. (2019). Efficacy of atropine 0.01% for the treatment of childhood myopia in European patients. Acta Ophthalmol..

[17] Diaz-Llopis M., Pinazo-Durán M.D. (2018). Superdiluted atropine at 0.01% reduces progression in children and adolescents. A 5 year study of safety and effectiveness. Arch. Soc. Esp. Oftalmol..

[18] Moriche-Carretero M., Revilla-Amores R., Diaz-Valle D., Morales-Fernández L., Gomez-de-Liaño R. (2021). Myopia progression and axial elongation in Spanish children: Efficacy of atropine 0.01% eye-drops. J. Fr. Ophtalmol..

[19] Loughman J., Kobia-Acquah E., Lingham G., Butler J., Loskutova E., Mackey D.A., Lee S.S.Y., Flitcroft D.I. (2023). Myopia outcome study of atropine in children: Two-year result of daily 0.01% atropine in a European population. Acta Ophthalmol..

[20] Lee S.S., Lingham G., Mforsci M.B., Sanfilippo P.G., Koay A., Franchina M., Chia A., Loughman J., Flitcroft D.I., Hammond C.J. (2022). Low-concentration atropine eyedrops for myopia control in a multi-racial cohort of Australian children: A randomised clinical trial. Clin. Exp. Ophthalmol..

[21] Yam J.C., Li F.F., Zhang X., Tang S.M., Yip B.H.K., Kam K.W., Ko S.T., Young A.L., Tham C.C., Chen L.J. (2020). Two-Year Clinical Trial of the Low-Concentration Atropine for Myopia Progression (LAMP) Study: Phase 2 Report. Ophthalmology.

[22] Gan J., Li S.M., Wu S., Cao K., Ma D., He X., Hua Z., Kang M.-T., Wei S., Bai W. (2022). Varying Dose of Atropine in Slowing Myopia Progression in Children Over Different Follow-Up Periods by Meta-Analysis. Front Med..

[23] Kaymak H., Graff B., Schaeffel F., Langenbucher A., Seitz B., Schwahn H. (2021). A retrospective analysis of the therapeutic effects of 0.01% atropine on axial length growth in children in a real-life clinical setting. Graefes Arch. Clin. Exp. Ophthalmol..

[24] Joachimsen L., Farassat N., Bleul T., Böhringer D., Lagrèze W.A., Reich M. (2021). Side effects of topical atropine 0.05% compared to 0.01% for myopia control in German school children: A pilot study. Int. Ophthalmol..

